# Information Technology in Radiation Oncology – A Brave New World?

**DOI:** 10.3389/fonc.2013.00116

**Published:** 2013-05-10

**Authors:** Martin-Immanuel Bittner

**Affiliations:** ^1^Department of Radiation Oncology, University Medical Center FreiburgFreiburg, Germany

The introduction of information technology (IT) into clinical practice has changed the way healthcare is being delivered. Electronic medical records (EMRs) replace paper records. In disciplines with high volumes of information to be processed, archived, and retrieved, the modern work scope would be difficult to navigate without the support of high capacity and secure servers, computers, and specialized robust software platforms.

Radiation oncology is an informatics based discipline that requires implementation of modern IT solutions. The use of modern informatics platforms has made possible vast progresses in radiation treatment planning over the last decade. In addition, radiation oncology is strongly dedicated to quality assurance issues. This is also an area where IT can help collect, audit, and archive the necessary data.

Kirkpatrick et al. (2013) describe the implementation of an EMR for a radiation oncology department as part of a large health enterprise. Their report outlines the process leading to the successful project completion, articulating reasons for the change as well as difficulties encountered of both technical and human nature.

The new system improved secure access to the department data both from intramural and extramural sources. This is one important advantage of IT solutions. Provided the hardware and software is available, data can easily be accessed and used simultaneously by several users at different places. In this case, clinical patient data from the Department of Radiation Oncology are also available to Emergency Department officers. In addition, there is a notable decrease in paper and stationary consumption, as well as needed archive space.

However, the system still includes paper-based components. Patient questionnaires and consent forms still have to be completed in a written form and scanned afterwards. The same is true for external documents which are to be available in the EMR. This may lead to error or limited efficiency.

Therefore, what are the next steps? Where will the future development migrate? The hybrid use of electronic and paper-based records is an initial strategy. The capabilities of modern mobile electronic devices and the variety of software solutions often freely available on the internet may alter healthcare delivery in the next decade. Will the ward-round be completed using a hand-held device with access to the patient’s data, being updated in real-time, and allowing for ordering of tests from the bedside? Is the radiation treatment planning going to be fully integrated in the digital workflow? Are quality assurance protocols part of an encompassing IT solution? Will data from EMRs directly be transferrable into clinical trials data capture systems, thereby dispensing of the need of redundant data entries?

All of these examples are currently in use in multiple institutions (Siochi et al., 2009; Patel et al., 2012; Yamamoto et al., 2012; Röhner et al., 2013). The integration of these informatics tools into one department will develop a new paradigm. A paperless, digital workflow, readily accessible to multiple clinicians and investigators, including interfaces to other departments’ and external healthcare providers’ data is at our horizon.

However, there are also considerations clouding this vision. How are sensible personal data secured? Who can view or alter them? Legal restrictions play an increasing role in working with personal data, as it is recognized that data protection will be a challenge moving forward. With more systems interconnected, a complete caption of patient characteristics and history are rendered possible. It has to be doubted whether our old concepts of data and data protection will remain valid given the potential of a fully digital environment. Concepts like the use of signatures for the verification of documents have to be reconsidered in a rapidly changing IT-driven world, where login details and user histories theoretically provide much more detailed information (Victoroff, 2012). Other hindrances include rivaling industrial standards which will have to be overcome to be able to exploit the full potential of integrated IT solutions within larger health networks (Takabayashi et al., 2011).

Kirkpatrick et al. (2013) report an important step on the way to an easier, safer, and more effective way to administer radiation oncology department data. However, the future of IT and electronic devices in healthcare has only just begun. It remains to be seen where it is heading.
